# High-Throughput Effect-Directed Analysis of Androgenic Compounds in Hospital Wastewater: Identifying Effect Drivers through Non-Target Screening Supported by Toxicity Prediction

**DOI:** 10.1021/acs.est.4c09942

**Published:** 2025-01-08

**Authors:** Iker Alvarez-Mora, Aset Muratuly, Sarah Johann, Katarzyna Arturi, Florian Jünger, Carolin Huber, Henner Hollert, Martin Krauss, Werner Brack, Melis Muz

**Affiliations:** † Department Exposure Science, 28342 Helmholtz Centre for Environmental Research—UFZ, 04318 Leipzig, Germany; ‡ Research Centre for Experimental Marine Biology and Biotechnology (PIE), University of the Basque Country (UPV/EHU), 48620 Plentzia, Basque Country, Spain; § Department of Evolutionary Ecology and Environmental Toxicology, 9173 Goethe University Frankfurt, 60438 Frankfurt am Main, Germany; ∥ Department of Environmental Chemistry, 28499 Swiss Federal Institute of Aquatic Science and Technology (Eawag), 8600 Dübendorf, Switzerland; ⊥ Department Environmental Media Related Ecotoxicology, Fraunhofer Institute for Molecular Biology and Applied Ecology (IME), 57392 Schmallenberg, Germany; # LOEWE Centre for Translational Biodiversity Genomics (LOEWE-TBG), 60325 Frankfurt am Main, Germany; ¶ Kompetenzzentrum Wasser Hessen, 60438 Frankfurt am Main, Germany

**Keywords:** high-throughput effect-directed analysis, nontargeted screening, chemical mixtures, hospital effluent, androgenic activity

## Abstract

The increasing number of contaminants released into the environment necessitates innovative strategies for their detection and identification, particularly in complex environmental matrices like hospital wastewater. Hospital effluents contain both natural and synthetic hormones that might significantly contribute to endocrine disruption in aquatic ecosystems. In this study, HT-EDA has been implemented to identify the main effect-drivers (testosterone, androsterone and norgestrel) from hospital effluent using microplate fractionation, the AR-CALUX bioassay and an efficient data processing workflow. Through nontargeted screening, over 5000 features (ESI+) were initially detected, but our workflow’s prioritization based on androgenic activity prediction reduced the number of features requiring further analysis by over 95%, significantly streamlining the workload. In addition, the semiquantitative nontarget analysis allowed for the calculation of the contribution of an identified compound to the total activity of the sample without the need for reference standards. While this contribution was low (∼4.3%) and applicable to only one compound (1,4-androstadiene-3,17-dione), it presents the first approach for calculating such contributions without relying on standards. Compared to the available alternatives our workflow demonstrates clear environmental relevance by enhancing HT-EDA for more efficient identification and prioritization of effect-drivers in hospital effluents, and it can be adapted to address other environmental threats in complex mixtures.

## Graphical abstract

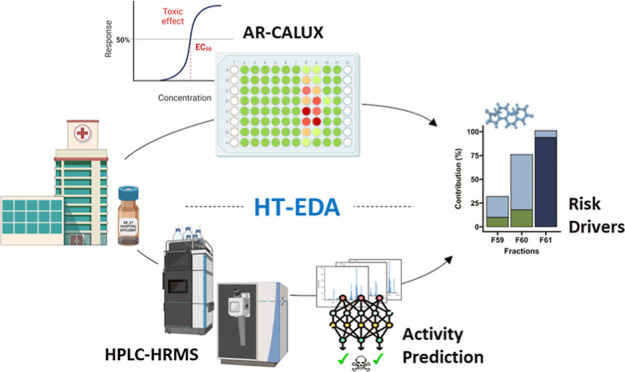

## 1 Introduction

The overwhelming number of contaminants released into the environment by everyday use and production requires novel strategies for their detection and identification in environmental matrices.^1^ While many of the single compounds may have limited impact on environmental health, some might pose significant risks, either directly or by contributing to complex mixture effects among which identifying toxicity drivers becomes challenging.^2^ Hospital wastewater is an example of these complex mixtures, capable of releasing pharmaceutical compounds with considerable potential for adverse environmental impacts.^3^ These effluents are frequently treated as equivalent to urban wastewater and sent to municipal treatment facilities, where conventional processes are not fully effective in eliminating contaminants.^4^ Particularly concerning are the endocrine-disrupting effects of hospital wastewater, primarily driven by natural and synthetic hormones used in various medical treatments and therapies.^5^

While new analytical methods can detect a wide range of the released compounds, chemical analysis alone does not offer insights into the sample toxicity. Therefore, combining analytical and bioanalytical techniques is vital to unravel the potential adverse effects of complex mixtures in the environment.^6,7^ Effect-based methods (EBMs) are small scale *in vitro* or *in vivo* bioanalytical tools that elucidate the mixture toxicity of samples consisting of both known and unknown compounds.^8,9^ These methods have started to be incorporated into regulatory frameworks (such as the water framework directive, WFD) for evaluating mixture risks, particularly in water bodies.^10^ However, water sectors and regulators are still concerned about the reliability and interpretation of EBM data.^11^ As regulation remains primarily compound-based, mainly because mitigation measures often rely on individual drivers, there is a need for strategies to prioritize these risk drives in complex environmental samples. Likewise, a thorough risk assessment requires more than just evaluating the activity of unknown compounds in an *in vitro* bioassay; understanding their adsorption, distribution, or metabolism is essential, and this necessitates knowledge of their identity. Effect-directed analysis (EDA) is one of the most promising tools for this purpose, integrating sample fractionation (e.g., by chromatographic separation) with bioassays and chemical analysis to focus identification efforts only on toxic sample fractions, containing fewer analytes.^12^ Although laboratory workloads significantly increase (instead of testing and analyzing one sample, many fractions have to be handled), testing and analyzing these fractions eventually helps to prioritize active compounds and consequently their identification. The large workload is, however, what hampers EDA application in large-scale studies, usually focusing on one or a few samples. Thus, high-throughput (HT) techniques encompassing sample preparation, biological analysis, and data processing are essential for EDA to manage the number of samples typically handled in monitoring studies.

HT-EDA enhances traditional EDA by combining microfractionation, high-throughput bioassays, and efficient chemical and bioanalytical data processing workflows, all with minimal manual intervention through extensive automation.^13^ The performance leap achieved through microfractionation into 96- or 384-well plates has been well demonstrated in recent studies thanks to their compatibility with EBMs in the same format.^14,15^ For bioassays to be compatible with HT-EDA, it is essential that they target highly specific, mainly receptor-based effects and demonstrate high sensitivity, as this enables a smaller number of contaminants to explain the observed effects (category 1 bioassays according to Escher et al. 2021 ^8^). So far, some of the endpoints that have been successfully implemented in EDA workflows using microfractionation include endocrine disruption [estrogenicity (ER), androgenicity (AR), progestogenic activity (PR) or thyroid hormone system disruption (FITC-T4 TTR-binding assay)],^16–18^ dioxin-like activity,^19^ or mutagenicity.^18^ Still, the primary bottleneck in achieving HT-EDA remains in the data processing step of nontarget screening (NTS). Despite the continuous development of tools for selecting significant features in HRMS files and aligning them, clustering isotopologues and adducts within each sample, and compensating between samples,^20^ the number of detected candidates continues to be high, even in fractions with retention time windows of a few seconds. Evaluating and identifying all these candidates is therefore a significant impediment to achieving HT-EDA, especially considering that most of them do not contribute to the specific activity being targeted.

The objective of this study was to develop a HT-EDA workflow to identify potentially androgenic compounds in a hospital effluent sample with high androgenic activity. This work contributes to the broader objective of the SOURCES project within the NORMAN network, which aims to identify source-specific contaminants across Europe. For that purpose, microplate-based fractionation using the Fractiomate was employed, and the androgenic activity of the extracts and fractions were assessed using the AR-CALUX assay.^21^ The main innovation of the study, however, was prioritizing relevant signals based on their MS/MS spectra with the MLinvitroTox^22^ toxicity prediction tool prior to compound identification, significantly enhancing the efficiency of this step. In this approach, potential toxicity was predicted before the bottleneck of structural elucidation and evaluation of candidate structures.

## 2 Materials and Methods

### 2.1 Sampling

To test the efficiency of the proposed workflow on an environmentally relevant sample, a wastewater sample from a hospital effluent was used. The sample was collected as part of the European SOURCES project of the NORMAN Network, in which it showed the highest androgenic activity among 124 samples from various sources (Figure S1). The sample was taken from the raw sewage of a hospital in the city of Berlin, Germany, by grab sampling 5 L of wastewater. The sample was stored in a PP bottle at −20 °C until extraction. A blank water sample was additionally prepared by transferring 200 mL of LC–MS grade water into a PP bottle and stored with the sample.

### 2.2 Sample Extraction

Following a protocol adapted from Välitalo et al.,^23^ previously evaluated for chemical and effect recoveries in Schulze et al.,^24^ both the sample and sample blank were filtered through 50 mm GF/F filters before extraction. For each liter of sample, 200 mg of HR-X SPE (solid phase extraction) cartridges (Macherey-Nagel, Germany) was used, equating to 1 g for the effluent sample. As reported in the reference study, scaling the extraction conditions does not affect the comparability across different extracted volumes. The solvent volumes for the conditioning and elution phases were also scaled according to the sample volume, using 5 mL of ethyl acetate, 5 mL of methanol, and 10 mL of LC–MS grade water per liter of sample as references, applied in that order. The sample (5 L) and blank (200 mL) were then extracted adjusting the flow rate to approximately 10 mL/min. The sample blank was prepared using the same extraction conditions as the sample to evaluate background contamination from the leaching of tubing and sorbents. After the extraction, the cartridges were rinsed with 5 mL of LC–MS grade water, and dried under nitrogen stream until dryness. For cartridge elution, the following solvent volumes were used as a reference per liter of sample: 2.5 mL of ethyl acetate, 2.5 mL of methanol, 2 mL of methanol with 1% formic acid, and 2 mL of methanol with 2% 7 N NH_4_OH. The extracts were evaporated under a nitrogen stream to reach a final relative enrichment factor (REF) of 1000 and stored at −20 °C until further analysis.

### 2.3 AR-CALUX Bioassay

The Chemically Activated LUciferase eXpression (CALUX) assay (AR-CALUX) was performed to evaluate androgenic activity of the sample and its fractions. The assay was performed on human osteosarcoma U2-OS cells purchased from Biodetection Systems (BDS, The Netherlands) transfected with a firefly luciferase reporter gene, coupled to the human androgen receptor (AR) as detailed in Sonneveld et al.^21^ and Wolf et al.^25^ Additionally, blank extract and fractions were also tested for quality control. Baseline activity was calculated as the median relative AR activity of the fractions (considering the mean of three technical replicates per fraction—Table S11). Fractions with modified *z*-scores greater than 10 were considered to have high androgenic activity

$$\mathrm{modified}\;z‐\mathrm{score}=\frac{X_{i}-M}{\mathrm{MAD}}$$

where *X*_*i*_—AR activity of the fraction *i*, *M* is the median AR activity of all fractions, MAD is the median absolute deviation, calculated as

$$\mathrm{MAD}=\mathrm{median}\left(|X_{i}-M|\right)$$

### 2.4 Fractionation

From the sample extract (REF 1000, 100% MeOH), a diluted aliquot of 10 μL (REF 200, 70:30 MeOH/H_2_O) was injected into the RP-HPLC system (Vanquish, Thermo Scientific). Chromatographic separation was achieved using a biphenyl column (Phenomenex, Kinetex 2.6 μm Biphenyl 100 A, 100 × 2.1 mm, equipped with a 5 × 2.1 mm precolumn of the same type and in-line filter), with a flow rate of 0.3 mL/min. The mobile phase consisted of water (A), methanol (B), both containing 0.1% formic acid to keep a pH of 2.7, and acetonitrile (C). The gradient elution program is shown in Table S1. Fractionation steps were performed using a FractioMate fraction collector (SPARKHolland & VU, The Netherlands). Fractions were collected every 18 s into 80 wells of a 96-well plate (24 min fractionation; the last 4.8 min of re-equilibration time were excluded), containing 10 μL of 5% DMSO in water used as a keeper. Fractionation was repeated twice, one for chemical analysis and one for biotesting. The microplates were dried under nitrogen flow at 45 °C, for approximately 2 h, and stored at −20 °C until further analysis. For chemical analysis, fractions were redissolved in 200 μL (REF 10) of MeOH/H_2_O (70:30). For biotesting, fractions were added to the exposure medium to achieve REF 50. To account for potential losses during chromatography and subsequent evaporation, a recombined extract was also prepared by placing the output of the analytical column into a test tube, and drying it under nitrogen flow. The fractionation method was optimized prior to use on the real sample using a mixture of 56 steroids and recoveries and potential RT shifts were assessed (Figures S2 and S3). No RT shifts were observed, so the Fractiomate starting delay was set to 0 s.

### 2.5 HPLC-HRMS Analysis

The unfractionated extract, blank, active fractions and calibration solutions of targeted compounds were analyzed in a Thermo Scientific Vanquish HPLC system coupled to a Thermo Scientific Exploris 480 quadrupole-Orbitrap mass spectrometer, equipped with a heated electrospray ionization source in both positive and negative ionization mode (HESI, Thermo Scientific, USA). To achieve a better ionization and improve the detection of steroid hormones, atmospheric pressure chemical ionization (APCI) was also used in the positive mode to complement HESI. To obtain chromatograms comparable to the toxicogram results from bioassays, the same chromatographic conditions used for fractionation were applied for the positive mode (see Section 2.4). In negative mode, the chromatographic gradient remained unchanged, but NH_4_F (1 mM) was used as mobile phase additive instead of formic acid. The mass spectrometer was operated in full scan–data dependent MS/MS (Full MS–ddMS/MS) with an intensity threshold of 8.0 × 10^4^ and a dynamic exclusion of 8 s. One full scan at a resolution of 120,000 full width at half-maximum (fwhm) at *m*/*z* 200 over a scan range of *m*/*z* 80–1200 was followed by 8 ddMS/MS scans at a resolution of 30,000 fwhm with an isolation window of 0.8 *m*/*z*. Further information regarding the ESI, APCI, and Full MS–ddMS/MS conditions can be found in the Table S2.

### 2.6 Data Processing

An overview of the data processing workflow is provided in Figure 1.

**Figure 1:**
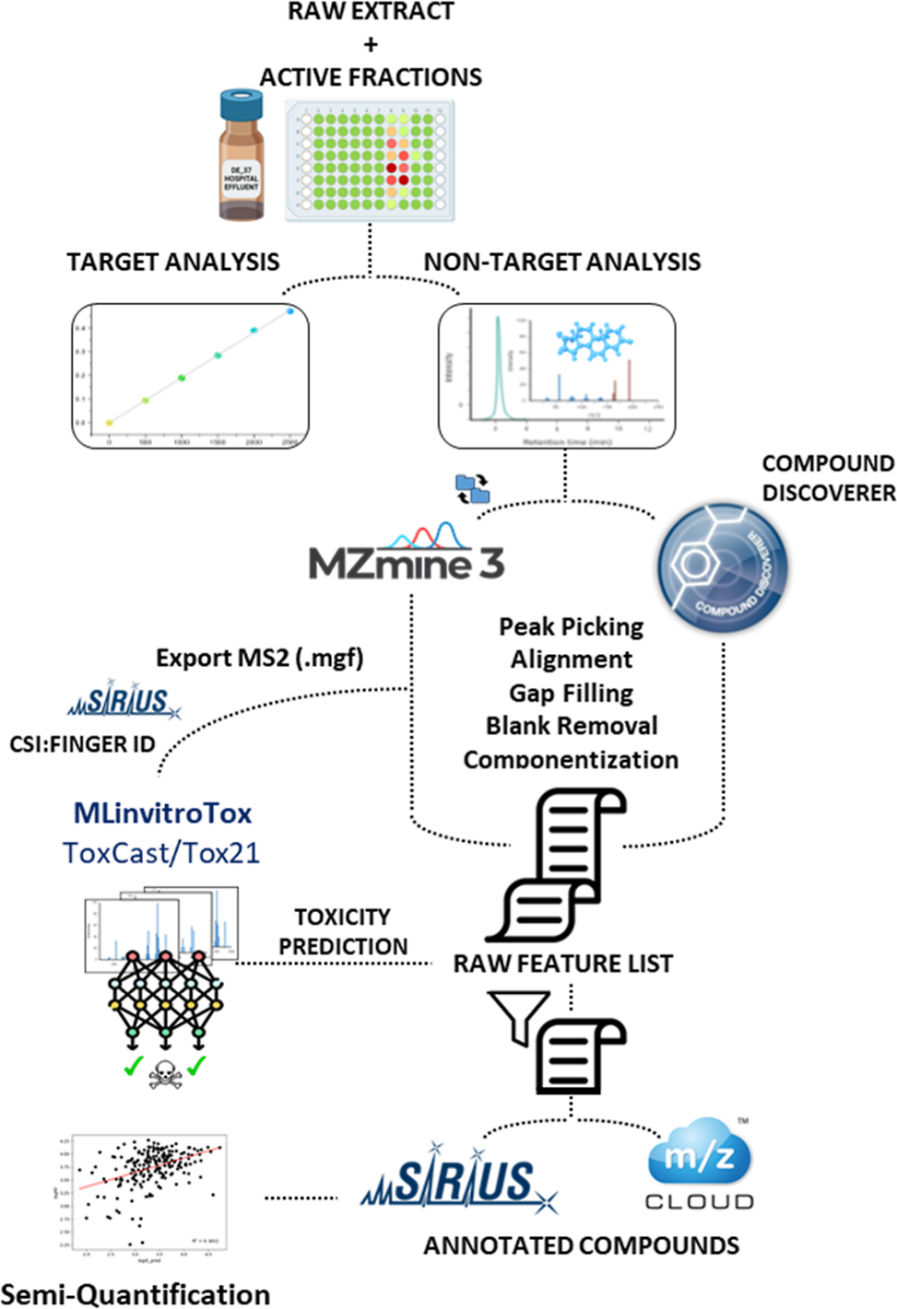
General workflow of the target and nontarget screening. MZmine and Compound Discoverer where used in parallel to process the data. MLinvitroTox was used to predict the toxicity of the features based on the MS/MS and filter the feature lists. Candidate annotation was based on spectral library search by mzCloud in the Compound Discoverer workflow and *in silico* prediction by SIRIUS in the case of the MZmine workflow.

#### 2.6.1 Target Analysis

The raw data (unfractionated extract, fractions and procedural blanks) were analyzed using vendor software TraceFinder 5.1 (Thermo) for the presence of 580 CECs typically occurring in waste and surface waters including pharmaceuticals, plant protection products, personal care products and industrial chemicals (Tables S3 and S4). The analysis included all of these compounds rather than focusing exclusively on (anti-) androgenic compounds, as the broader project aims to assess a wide range of micropollutants. For hormonal steroids, a second method was employed to evaluate the APCI data, where the [M + H]^+^ or [M – H_2_O]^+^ was selected as the quantification ion, depending on which was more suitable. A maximum mass error of 5 ppm was set for peak identification. The acceptance criteria for each compound included passing the isotopic profile flag, the fragmentation flag, and falling within the established retention time window (adapted for each compound). Twelve calibration points in the range of 0.1–750 μg/L were analyzed with the samples. When redissolving the samples, in addition to the calibration points, a mixture of isotopically labeled internal standards was added to have a final concentration of 50 μg/L to correct peak areas for potential instrumental fluctuations and batch effects (Table S5).

#### 2.6.2 Nontarget Screening

The HPLC-HRMS (ESI+ and APCI+) data originally in raw format was converted to .*mzML* format using the msconvert tool,^26^ with vendor peak-picking for centroiding. The data was then imported into the MZmine 3.9.0 software for data processing.^27^ The complete workflow of the data processing steps and their parameters can be found in the Table S6 in Supporting Information. Since fractionation restricts the maximum REF achievable in the fractions (REF 10), this strategy was implemented on the sample and blank extracts (REF 50) to ensure that low-intensity features were not missed. However, it was also subsequently verified whether these features were present in the fractions.

The resulting *.mgf* files were imported to SIRIUS 5.8.6 software for calculation of molecular fingerprints.^28^ The molecular fingerprints were saved as compressed files, and resulting file structure was used for prediction of androgenic activity using the MLinvitroTox workflow, as described in Arturi and Hollender.^22^ Briefly, in MLinvitroTox, supervised machine learning XGBoost classifiers with SMOTE (Synthetic Minority Oversampling Technique) were trained on SIRIUS molecular fingerprints to predict nearly 400 target-specific and 100 cytotoxic endpoints from ToxCast and Tox21 databases. The relevant models for androgenic activity (i.e., reporter gene assays TOX21-AR-BLA Agonist-ratio and TOX21-AR-LUC-MDAKB2 Agonist, and the Nuclear translocation assay UPITT-HCI-U2OS-AR-TIF2 Nucleoli-Agonist) were selected and used to predict the potential bioactivity of the features in the sample extract and its fractions. Keeping a conservative approach, any hit (hit probability >0.5) in at least one of the three endpoints was considered as potential active feature. Due to the poor model performance in the negative mode, the prioritization based on toxicity prediction was only conducted for data acquired in the positive mode. This list of prioritized features was used to filter the feature lists, based on their *m*/*z* values with tolerance of 5 ppm, and retention times with tolerance of 30 s. Structure elucidation was performed by fingerprint-based identification using CSI:FingerID (integrated in SIRIUS).^29^ The molecular formula identification was performed with a mass accuracy of 5 ppm, using the De novo and bottom-up approach for molecular formula generation (all parameters are shown in Figure S4). Hereby, structural databases, such as KEGG, PubMed, and NORMAN-SUSDAT were searched for matches to the predicted molecular fingerprints, leading to level 3 identifications.^30^ In general, matches with a Tanimoto similarity of greater than 80% and a CSI:FingerID Score of less than −70 were accepted, although spectral matching was ultimately subject to manual verification.

In parallel to the workflow in MZmine, the Compound Discoverer (3.3) workflow shown in Figure S5 was used to streamline identification of features prioritized by MLinvitroTox in the MZmine workflow. For further identification, the peak shape of the candidate features was assessed (peak rating >6 and manually evaluated) and only features with acquired MS/MS were considered. The structural elucidation was based on the comparison of experimental MS/MS spectra with the mzCloud spectral library. To aid the identification of tentative candidates when the spectral library search was not successful, the NORMAN SUSDAT^31^ and ChemSpider mass lists were used for providing potential candidates to reinforce those identified at level 3 using SIRIUS (as explained above). The annotation of identified candidates was done according to the confidence levels suggested by Schymanski et al.^30^ Only candidates within levels 2 and 3 of confidence were reported (i.e., candidates with spectral library hit or potential structures matching *in silico* prediction, respectively).

The candidates with an assigned probable structure were semiquantified using a modified methodology based on the work by Sepman et al.^32^ with slight differences (e.g., ECFP—extended connectivity fingerprints—were also included^33^). This method was limited to the positive ionization mode due to model performance limitations for the negative mode, so it was only applied to this ionization mode. For the semiquantification of candidates with spectral hit from mzCloud (level 2a), the molecular fingerprints of the candidates were calculated from their SMILES strings using RDKit 2024.03.2 and CDK 2.9. For the tentatively identified compounds (level 3), molecular fingerprints were predicted from experimental MS/MS spectra in SIRIUS and then used to predict ionization efficiency, since each candidate has its own SMILES.

To compare the efficiency of the proposed workflow with previously available approaches, the sample was reprocessed using Compound Discoverer in parallel, without prioritization through MLinvitroTox. The detected features list was reduced by filtering based on retention time (15–20 min), minimum intensity (>10^6^), and peak rating (>6). The filtered list was manually inspected to exclude peaks that, despite passing the filter, did not exhibit an acceptable peak shape. The candidates retrieved from mzCloud and the masslists for the remaining features were assessed for independent androgenic activity prediction using VEGA QSAR.^34^ The final candidates were compared to those obtained from the workflow using MLinvitroTox. The same workflow was also applied for the negative mode, since the MLinvitroTox prediction models for [M – H]^−^ generally showed poor performance.

### 2.7 Contribution of Identified Compounds to Fraction Activity

The results from the (semi)­quantification and dose–response curves were used to assess the contribution of the compounds based on iceberg modeling.^8^ Relative potency values (REP) reported in the literature were used for these estimates, or EC50 values were employed to calculate the REP as detailed in Supporting Information.2.

## 3 Results and Discussion

### 3.1 Androgenic Activity of the Hospital Effluent Extract and Its Fractions

The androgenic activity of the unfractionated and recombined extracts was measured with the AR-CALUX bioassay. The concentration response curves were normalized to the response of dihydrotestosterone (DHT), and fitted into a sigmoidal model against the enrichment factor (Figure S6). The effective concentration to achieve 10% of the maximum response (EC10) for unfractionated and recombined extracts were relative enrichment factors (REF) of 0.052 and 0.027, respectively. DHT equivalents were calculated as 278.9 ng DHT/L and 291.3 ng DHT/L for unfractionated and recombined extracts, respectively. The AR activity of the recombined extract was nearly 2-fold higher than the unfractionated extract, which could be attributed to normal variability in the bioassay. Given the decrease in activity at the highest REF tested, a second approach was used to calculate the EC_10_ using the linear range of the dose–response curve at the lowest concentrations (Table S11). Although the exact value varies slightly, the differences (EC_10_ = 0.07 and 0.03 respectively) remain.

After fractionation, 80 fractions were tested with the AR-CALUX assay to identify the active ones at a REF 50, which was the highest enrichment factor that could be tested in the fraction screening. Ten fractions were found to have quantifiable androgenic activity, with modified *z*-scores ranging from 18.8 to 97.0 (Figure 2). The fractions identified as having high androgenic activity were labeled as B8, C8, D8, E8, F8, G8, C9, D9, E9, and F9, according to their position in the 96-well plate. The fractions of the respective blank sample did not show any significant activity (Table S7). The toxicogram presented in Figure 2 reveals two distinct peaks of androgenic activity. The first peak occurs between 15 and 16.5 min with fractions B8 to G8. The second peak appears between 17 and 18.5 min, with fractions C9 to F9.

**Figure 2:**
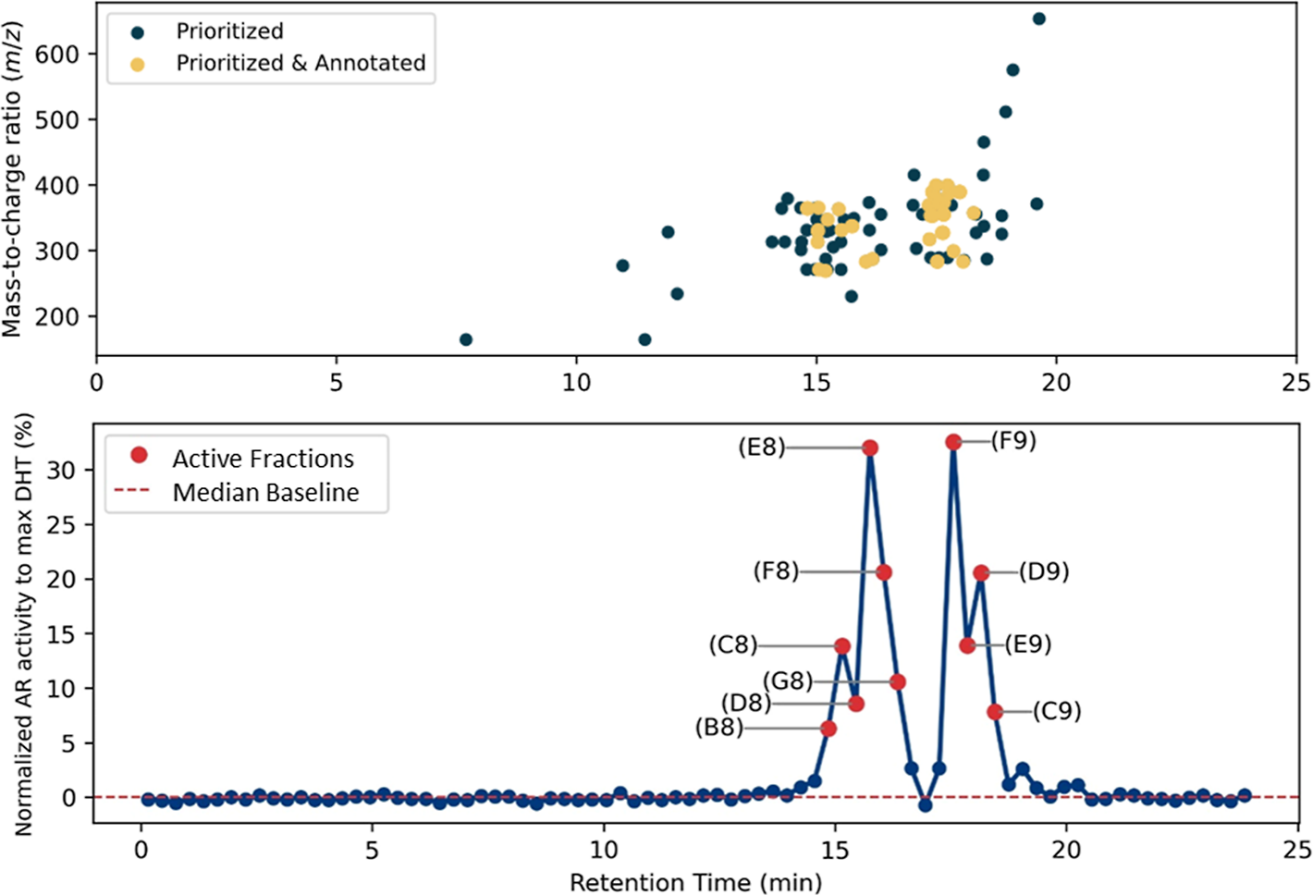
Toxicogram showing the relative AR activity of the fractions of the hospital effluent extract tested at REF50 (down) and overlapping of the toxicogram with the features prioritized and annotated in the NTS (top). AR activity of each fraction was normalized to the maximum reference DHT activity. AR-CALUX was performed on 96-well plates.

### 3.2 Compounds Revealed from Target Screening

From more than 500 analyzed CECs, 26 compounds were found using targeted analysis in the retention time window of active fractions, including various classes of CECs, such as pharmaceuticals, industrial chemicals, etc. As shown in Figure 3, detected compounds were classified in four application categories, known androgens, pharmaceuticals, disinfectants and industrial chemicals and additives. Detailed information about concentrations in the unfractionated extract and fractions, RT and method used for the quantification of these compounds can be found in Table S8.

**Figure 3:**
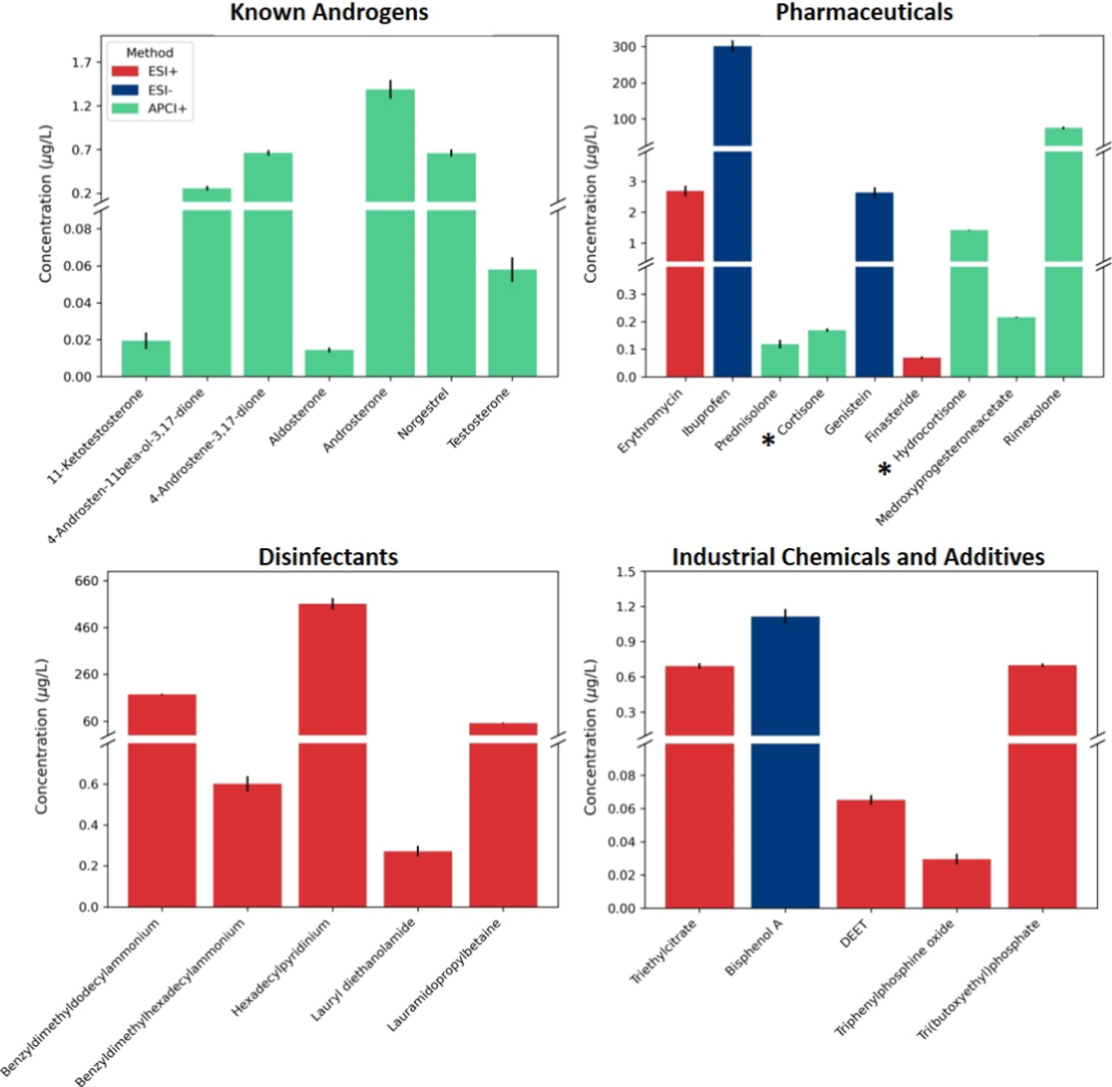
Concentration of targeted chemicals in the unfractionated extract, categorized as known androgens, pharmaceuticals, disinfectants, and industrial chemicals and additives. Bar colors indicate the ionization mode used for quantification. * Natural glucocorticoids also used as pharmaceutical drugs.

As expected, several chemicals commonly used in large quantities in hospitals, such as disinfectants, were detected at high concentrations. For instance, hexadecylpyridinium was found at a concentration of 561 μg/L and benzyldimethyldodecylammonium at 173 μg/L. The high concentrations found for these compounds are above the highest calibration point analyzed and therefore their correct quantification cannot be guaranteed and are reported as >20 μg/L. Similarly, several commonly used pharmaceuticals, such as ibuprofen and rimexolone, were detected at high concentrations (>20 μg/L). This result was expected given the source of the sample. While these compounds may not directly relate to the detected androgenic activity, they are important for the project’s broader goal of identifying source-specific contaminants. This is particularly evident for detected pharmaceuticals or disinfectants. For instance, compared to the study by Finckh et al.,^35^ concentrations were found to be about an order of magnitude higher than those in conventional WWTP effluents sampled across Europe. Additionally, disinfectants that were undetectable in these WWTP effluents were also found in the hospital effluent.

The miscellaneous category included a variety of compounds, such as bisphenol A and tri­(butoxyethyl)­phosphate, mainly used in the production of plastics, that had the highest concentration of 1.1 and 0.7 μg/L, respectively. Other compounds in this category include insect repellents (DEET) and food additives (triethylcitrate). None of these compounds were reported to have AR agonistic activity. On the contrary, bisphenol A was identified as an antagonist of AR signaling.^36^

Seven known androgens were quantified in the unfractionated extract: Norgestrel, 11- ketotestosterone, aldosterone, 4-androsten-11β-ol-3,17-dione, androsterone, 4-androstene-3,17-dione, and testosterone (Table S8). Among them 4-androstene-3,17-dione, androsterone, and norgestrel had the highest concentrations of 0.67, 1.39, and 0.66 μg/L, respectively. Testosterone, the most potent androgen among the compounds quantified, was found in lower concentration of 0.06 μg/L. Norgestrel is produced synthetically for its use in medication to treat hormonal deficiency or as a contraceptive, and was previously reported to have androgenic activity.^37^ Testosterone and 4-androstene-3,17-dione are endogenous, but also used as medications, while the rest are primarily endogenous hormones. All these steroids are commonly found in human excretions, and their presence in the sample is expected.^38,39^

### 3.3 Nontarget Screening Results

#### 3.3.1 Prioritization and Identification of Effect-Drivers in NTS Supported by MLinvitroTox

A nontargeted analysis was conducted to identify potential effect-drivers within the active RT window that might have been missed by the targeted analysis. Mass spectra from both APCI-MS and ESI-MS were used to identify compounds using Compound Discoverer and SIRIUS. The results of the nontargeted analysis for confidence levels 2a and 3 are shown in Table S9. Initially more than 5243 features were detected after blank subtraction (peak intensity in samples >300% of procedural blanks) in the active RT window by ESI+ and 1469 by APCI+ (3016 and 724 with assigned MS/MS data spectra respectively). After the prioritization of the features based on the predicted AR activity by MLinvitroTox, only 83 features (ESI+) and 53 features (APCI+) were selected for further analysis, with 25 features shared for both lists. Thus, prioritized features constituted only 1.6% and 3.6% of total number of features, respectively, significantly reducing the workload for further structure elucidation (Figure S7). In turn, Figure 2 shows a good overlap between the prioritized features and the activity peaks of the toxicogram. After prioritization, 13 features were annotated using ESI-MS data through reference spectra matches or *in silico* fragmentation matches. Additionally, 11 features were annotated using APCI-MS data, with five features being common to both methods.

From these, eight compounds were identified at confidence level 2a (four solely by ESI-MS, three solely by APCI-MS, and one by both methods). Additionally, nine features (ESI-MS) and eight features (APCI-MS), respectively, were annotated with confidence level 3, with 2–5 probable structures for each feature (Table S9). Therefore, 28 more prioritized (including seven annotated) features were identified only by APCI+, which means, it allowed significantly increasing the detectable chemical space. It is especially useful for the identification of steroid hormones since APCI is often the preferred method for their detection in complex matrices, providing lower limits of detection and reduced matrix effects.^40^

At level 2a, the identified compounds were mainly steroid hormones, and their metabolites. These include progesterone, cortisol, DHEA, and various hydroxylated and oxo-derivatives. Cortisol metabolites (cortolone, 20β-dihydrocortisol, and 6β-hydroxycortisol) were the largest group of identified steroids. Cortisol is a primary glucocorticoid hormone, and it plays a role in stress response and metabolism. Even though there is no direct evidence of cortisol being an androgen, it regulates the metabolism of androgens, and can alter androgen levels indirectly.^41^ As the cortisol is an endogenous hormone, it is likely that it is present in the sample due to the presence of human excretion.^42^ Additionally, cortisol can also be supplemented in the form of the medication of hydrocortisone, so the origin of the compound is likely to be both endogenous production and medication. Similarly, progesterone and DHEA are essential sex hormones, and their presence in the sample is consistent with their natural occurrence, as well as the widespread use in medication. Among the compounds identified at level 2a, DHEA (REP not available) and 1,4-androstadiene-3,17-dione (REP = 1.2 × 10^–3^)^38^ were compounds with known androgenic properties.^41,43^ Consequently, the contribution calculation will focus solely on the latter, as no AR CALUX potency data is available for DHEA.

At level 3, steroid hormones and their metabolites were the most common group of compounds including progesterone and testosterone hydroxylated and ester derivatives. Some probable structures also included bile acids, such as nutriacholic acid, and cholic acid, as well as their derivatives. These compounds are human bile acids, produced in the liver and, secreted into the bile. Some of them, such as brexanolone and adrenosterone and one surfactant, annotated as either dodecyldimethyl hyroxylammmonium (DDAC) or lauramine oxide (LDAO), were also identified at this level. Both of these compounds are common antimicrobial surfactants, used in cleaning products. Interestingly, DDAC was previously reported to have androgenic activity,^44^ while LDAO was reported to be inactive.^43^ Although the prioritization with the toxicity prediction could guide us toward one candidate, the MS evidence does not allow us to distinguish between them. To remain cautious, we have chosen to report both possibilities.

In summary, in addition to the 7 compounds quantified by the dedicated target screening, a total of two compounds with reported androgenic activity (DHEA and 1,4-androstadiene-3,17-dione) were annotated by the NTS workflow at level 2a. In addition, at least one candidate with reported androgenicity (DDAC) was identified at level 3 among other potentially active metabolites that also deserve attention. The negative mode analysis also identified additional candidates, mainly metabolites of previously identified compounds, which, although not prioritized by the toxicity prediction tool, may require further investigation.

To provide quantitative information about the identified compounds, semiquantification was performed in the unfractionated extract, according to the method described in Section 2.6.2. For this purpose, SMILES strings of the compounds, identified at confidence level 2a, and SIRIUS generated molecular fingerprints of the compounds identified at confidence level 3, were used to predict the ionization efficiency, and consequently, the response factor of compounds. The results of the semiquantification for the unfractionated extract are also shown in Table S9.

Although prioritization of compounds by MLinvitroTox was not applied for the negative ionization mode due to bad model performance, an NTS was performed following the Compound Discoverer workflow described in Section 2.6.2. As shown in Table S10, 27 compounds were identified between levels 2a and 2b, and 13 more features were annotated as level 3. The QSAR prediction identified three potential effect-drivers, all of which are natural products: xanthohumol, the glucocorticoid cortisol and steroid metabolite 2-methoxy-estradiol-17beta 3-glucuronide. Additionally, many metabolites of compounds quantified or prioritized in the second peak of activity (minutes 17–19) were present. Some of them, such as glucuronide conjugates of testosterone, DHT or androsterone, elute at retention times corresponding to fractions F9, G9 and H9. Some of them such as 11-oxo-androsteroneglucuronide, 5-α-androstan-3α,17β-diol glucuronide or 11-beta-hydroxyandrosterone-3-glucuronide were also detected at retention times corresponding to the peak of activity between minutes 15 and 16.5.

#### 3.3.2 Comparative Assessment of Workflow Efficiency

The improvement in the proposed workflow’s performance is evident with the incorporation of the unique tool capable of making predictions based on MS/MS data (Figure S7). However, to evaluate its strengths and weaknesses and to demonstrate the advancement this workflow brings to HT-EDA compared to alternatives, it was compared to a workflow that includes toxicity prediction after structure elucidation. Such workflows, using QSAR modeling to predict the activity of final candidates, have been widely used in EDA previously.^16,45^ The candidates annotated through the Compound Discoverer workflow without applying MLinvitroTox, as well as the predictions using VEGA QSAR for all candidates, are shown in Table S9A,B.

The first evident difference between the two workflows is the applied filters. In the workflow without prioritization, more stringent filters are used to handle the large number of detected features, which may lead to false negatives. This resulted in a filtered list with 1233 features that were manually inspected to exclude peaks that, despite passing the peak rating filter, did not exhibit an acceptable peak shape. The same process was done for the 83 prioritized features in the proposed workflow. After this stage, 378 features were considered for retrieving the structures of potential candidates for activity prediction (1107). SMILES were obtained from EPA’s CompTox database, requiring data curation, as not all compound names retrieved from Compound Discoverer matched entries in CompTox.

After the predictions, 61 unique active structures were identified, leading to the annotation of 15 features at levels 2a and 3. Four compounds were annotated at level 2a, three of which overlapped with the five compounds annotated in our workflow (see Section 3.3.1). The remaining two were predicted as inactive (low reliability) by QSAR VEGA. For the level 3 annotations, most features prioritized by VEGA but not by MLinvitroTox also had some candidate structures predicted as inactive or active with low reliability, raising the possibility of false positives. However, it appears that there are two active features (good/experimental reliability) that were not prioritized by MLinvitroTox. Conversely, some features prioritized by MLinvitroTox were not prioritized in this second processing due to failing the stricter filter that were necessary to manage a more feasible number of features. Within the uncertainties inherent to each model, the proposed workflow with MLinvitroTox avoids false negatives arising from filters applied to the initial feature list and bottlenecks such as peak inspection or irrelevant candidate structure evaluation. However, applying structure-based QSARs proves highly valuable for discriminating between candidate structures in features annotated as level 3. Given the clear advantages in reducing data processing workload and the comparable performance of MS/MS-based predictions over structure-based predictions, the proposed workflow represents a significant advancement in HT-EDA.

### 3.4 Contribution of Identified Compounds to the Total Androgenic Activity of the Hospital Effluent

The contribution of identified compounds to fraction activity was estimated using iceberg modeling,^8^ assuming that the total bioactivity is explainable by the sum of the activity of each of the contributors. In EBM studies without fractionation, contribution is calculated using the total activity and concentrations of the unfractionated extract, overlooking possible antagonistic effects. HT-EDA studies can unmask these effects through fractionation and often use concentration–response curves of active fractions to explain contributions. However, measuring the dose–response curves of the fractions to calculate contributions to overall activity also requires calculating concentrations of the compounds in those fractions in addition to those in the unfractionated extract.

Despite the additional work involved, the contribution of the activity was studied on each fraction independently, using the concentrations measured in each fraction specifically. Compared to the approach described by Jonkers et al.,^14^ our method offers two main advantages, though it is more time- and labor-intensive. First, it allows us to account for the activity contributions of individual fractions, helping to reveal potential masking effects. Second, it provides a more accurate estimate of the concentration in each fraction, as it considers potential losses during fractionation (differences between calculating concentration in the fraction or the unfractionated extract can be observed in Table S9). Thus, if a quantitative explanation of the identified compounds’ contributions to the observed activity in each fraction is needed, this approach is essential. Conversely, if the aim is a more qualitative assessment for identifying effect-drivers, the Jonkers et al. approach offers a more efficient alternative.

As described in Section 3.2, all the potentially androgenic compounds covered by the target analysis elute in the second active region. To reduce the additional workload associated with optimizing a new chromatographic method tailored to these fractions, the identical method employed for the unfractionated extract was utilized, given its demonstrated efficacy in separating the target steroid hormones. From each of the two activity peaks shown in Figure 2, three fractions were selected—D8, E8 and F8 from one peak and F9, D9 and E9 from the other—giving a total of six fractions for testing concentration–response curves by a dilution series. Figure 4 displays only F9, E9, and D9, as the rest did not reach 10% activity in the studied REF range of 0–16, and therefore the concentration–response curves were not robust enough to derive EC10 values (Table S11). Although the relative activity appears similar at REF 50 for both peaks, cytotoxicity effects at high REFs in gene-reporter assays often flatten or reduce the effect curve around 30–50% activity.^8^ The results from the dose–response curves of the fractions indicate that the activity of fractions D8, E8, and F8 is negligible compared to D9, E9 and F9. To verify this, note that the EC_10_ of fraction E9 (the least active among the measured fractions) is REF 0.39. Since the EC_10_ values for D8, E8, and F8 are all higher than REF 16 (the highest tested REF), their activity would not exceed 2% of E9’s activity. The NTS results indicate that the two activity peaks may correspond to the parent androgen hormones (peak 2, RT 17–18.5 min) and some of their respective metabolites (peak 1, RT 15–16.5 min), such as 11-oxo-androsterone glucuronide, 5-α-Androstan-3α,17β-diol glucuronide, or 11-beta-hydroxyandrosterone-3-glucuronide. While these phase II metabolites are not bioactive, deconjugation can occur during the sample preparation process, releasing the bioactive parent compound. Even if the yield of this deconjugation to active metabolites is low, the much higher concentration of the metabolites (e.g., the peak area of testosterone sulfate is 100 times larger than that of testosterone) could be sufficient to account for the measured activity.

**Figure 4:**
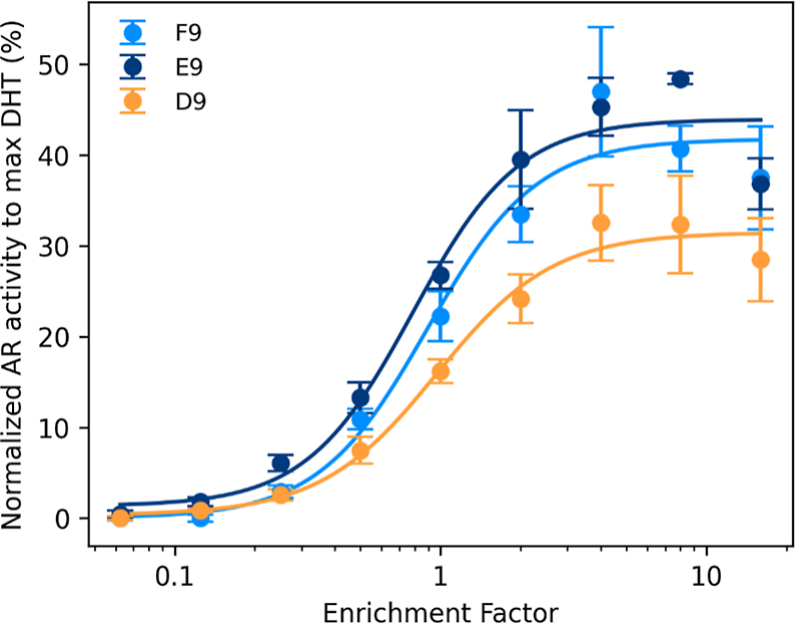
Concentration–response curves of the toxic fractions. AR activity was normalized to maximal response of DHT and fitted to a sigmoidal curve. Error bars represent the standard deviation of three technical replicates. AR-CALUX was performed on 384-well plates.

BEQ_bio_-s for each fraction were derived from their EC_10_ value. The bioanalytical equivalent concentrations were 0.05, 0.06, and 0.049 μg DHT/L for F9, E9, and D9, respectively, aligning well with the BEQ values calculated by the BDS evaluation approach (Table S11). Three known androgenic compounds with known EC10/50 or REP values, i.e. testosterone, androsterone and norgestrel, were used to calculate the contribution to these fractions. In fraction F9, testosterone and androsterone were detected in concentrations, accounting for 16 and 34% of the activity of the fraction respectively (Figure 5A). These two compounds elute between the latter fraction and E9 in which they also account for 3% and 11% of the bioactivity, respectively. Finally, for D9, androsterone was still detected in low concentration, accounting for 9% of the activity, and norgestrel could explain 226%.

**Figure 5:**
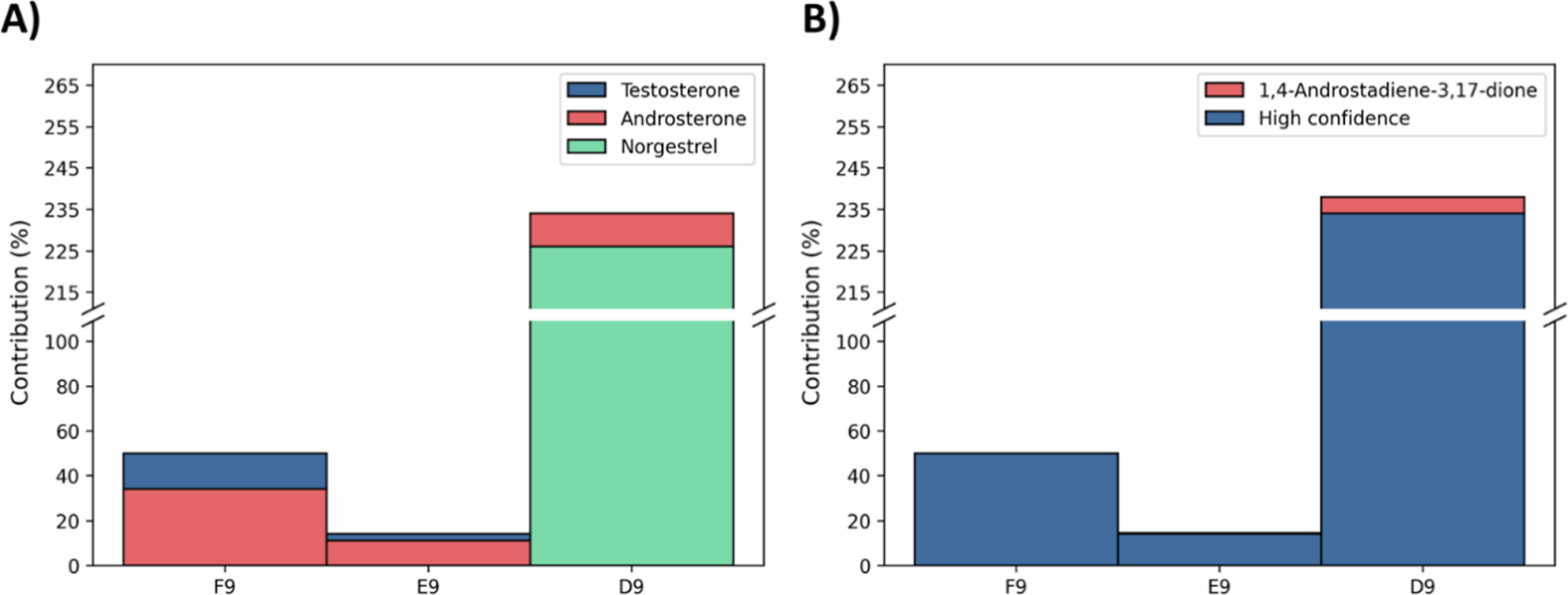
Contribution of detected compounds to the total AR activity with high level of confidence (A) and including medium level confidence results (B).

The activity overestimation in fraction D9 might be due to the presence of antiandrogenic compounds in the same fraction. Among the compounds quantified in the target screening, we identified several known antiandrogenic substances, including bisphenol A, finasteride, and medroxyprogesterone acetate.^46–49^ Notably, finasteride and medroxyprogesterone acetate elute within the peak activity window (RT = 17.78 and 19.02 min, respectively), potentially influencing the calculation of activity contribution in these fractions. Moreover, REP values were not experimentally measured in this study, but were taken from other studies using the same bioassay, which may also be the reason for this overestimation, but significantly increases the throughput of the HT-EDA workflow. Experimental confirmation would be needed to confirm the REP of this compound in particular.

Overall, the results indicate that both natural and synthetic hormones present in hospital effluent are the contributors to the androgenic activity observed in the sample. Compared to urban wastewater, where according to Almazrouei et al.,^5^ the concentrations of these hormones rarely exceed a few ng/L, hospital wastewater releases significantly higher concentrations, making it a major source of these compounds in surface waters. In turn, identifying that androgenic activity from hospital effluents is linked to a few compounds with similar structures and properties could guide remediation efforts toward targeting and removing these specific substances.

Additionally, the contribution of the compounds, identified by NTS at confidence level 2a using ESI+, was considered employing semiquantification results. Only 1,4-androstadiene-3,17-dione had available information on its activity in AR-CALUX. The semiquantified concentrations for this compound in E9 and D9 were 0.17 and 1.4 μg/L, respectively, and its REP value was 1.2 × 10^–3^.^38^ Therefore, the contributions of this compound to the activity of the fractions were calculated to be 0.34% and 4.3%, respectively. Although the explained contribution is not very high, this is the first time that semiquantification of compounds identified by NTS has been used to explain their contribution to fraction activity in HT-EDA. The added value of this approach is currently limited and requires further investigation, particularly in combination with MS2-based toxicity prediction, which has proven more informative. This integration could ultimately enhance the estimation of activity contributions beyond known compounds. The difference in explained contribution between the target and nontarget analyses is evident. Although identifying the precise cause is challenging, three possible explanations can be considered: (i) the main activity drivers are the compounds included in our target list, with the unknowns contributing only a small fraction to the activity in this specific sample, (ii) due to higher uncertainty in the semiquantification method, the activity of the unknowns may be underestimated, or (iii) the NTS workflow may overlook certain active compounds. These possibilities warrant further investigation in future studies.

It is also important to distinguish the level of confidence with which we can report this contribution, as the semiquantification results are not as reliable as targeted analysis, mainly because it is still difficult to estimate their uncertainty. Alvarez-Mora et al.^13^ suggested that the explained contribution by semiquantification results should be reported with a medium level of confidence, while the contribution confirmed by targeted analysis using the reference standard should be reported with a high level of confidence (Figure 5B). Semiquantification results could also be used to estimate the contribution of these compounds to the total AR activity, thus prioritizing the most promising compounds that can be later tested for confirmation.

### 3.5 Novelty, Limitations and Future Direction of the Proposed Workflow

Since the development of fraction collectors designed for microplates compatible with high-throughput bioassays, attempts have been made to develop EDA toward applicability in monitoring. However, the primary bottleneck has been identifying toxicity drivers using NTS workflows. Our study aims to bridge this gap by using a prioritization tool that directs elucidation efforts toward features relevant to the study’s endpoint. The workflow demonstrated that prioritization based on predicted AR agonist activity can significantly increase the throughput of nontarget screening by reducing the number of features that need to be identified from thousands to tens (Figure S7). Since the machine learning model for toxicity prediction used molecular fingerprints that can be computed from MS/MS data for training rather than structure of chemicals, prioritization can be performed before the structure elucidation. Until now, some QSAR-based toxicity prediction tools have been used to prioritize compounds in EDA, but always following their prior structural elucidation. HT-EDA can, on the one hand, greatly benefit from tools like MLinvitroTox, and on the other hand, these studies are ideal for testing the potential of such tools, as they can be used to prioritize potentially active features in combination with bioassays rather than to replace them. An important consideration for applying this approach is the need for high-quality MS/MS spectra for the features in each fraction.^22^ This may require rerunning the fractions with a dedicated inclusion list to improve MS/MS spectral coverage of the MS1 peaks. An important advantage of MS/MS-based predictions is that they predict the activity of the feature itself rather than the candidate structures within each feature. This is particularly valuable for compounds annotated at level 3, where multiple structures may fit within a single feature. QSARs could introduce false positives if we were to prioritize structures predicted as active over other candidates. Therefore, using both tools complementarily would be a highly effective approach. For instance, if a feature prioritized by MLinvitroTox has three possible structures, with only one predicted as active by QSAR, this could serve as evidence to increase the confidence level of the annotation.

One possible reason for the overestimated activity attributed to norgestrel could be the presence of antiandrogenic compounds eluting within the same fraction. Therefore, screening fractions for antiandrogenic activity is highly recommended in studies of this kind, even though it was not feasible in the present study. An alternative recommendation arising from this study is to incorporate antiandrogenicity predictions into the workflow, which should be assessed in future research. Furthermore, as toxicity prediction enables the visualization of potentially active compound distribution in the chromatogram effortlessly, this information can guide further refinement of separation in HT-EDA.

Despite the significant prioritization potential of the workflow, some weaknesses indicate that further work is needed. For example, the activity prediction currently only functions efficiently in positive ionization mode. As mentioned earlier, the study aims for high throughput without compromising on comprehensiveness and robustness, which led to using two different ionization sources and analyze the fractions separately. For this reason, the same workflow should be adaptable to ensure efficiency in negative ionization mode as well. While not addressed in this study, the chromatographic conditions employed for the analysis of the fractions can also be adapted to align with the elution time of the active compounds. By focusing the optimization of the separation within a specific chromatogram window, the chromatographic resolution can be enhanced and the potential for interference between coeluting peaks can be reduced.

Beyond that, it is clear that NTS workflows still heavily rely on spectral libraries, and for many unmatched features, elucidating their structure through fragmentation prediction with CSI:FingerID in SIRIUS was not possible. Similarly, semiquantifying approaches continue to be a source of contention with regard to their efficacy. While this is beyond the scope of the present paper, it would be beneficial to further validate their efficacy when used to estimate the contributions of effect-drivers. Thus, far, it appears that the models based on ionization efficiency prediction are the most robust. Within these, there seem to be differences between the use of descriptors such as molecular graphs or fingerprints derived from MS2 spectra or structures. An evaluation of their efficacy in EDA studies could therefore be the next step.

The workflow presented in this study integrates microfractionation with downscaled gene-reporter assays and incorporates a novel NTS approach including advanced tools for prioritization and semiquantification, maintaining a high level of efficiency for robust HT-EDA. The study confirmed that the main drivers of androgenicity in hospital effluents are both natural and synthetic hormones, specifically testosterone, norgestrel, and androsterone in our case. Additionally, the tools employed, such as toxicity prediction and semiquantification, are not limited to androgenicity but can be applied to a range of other endpoints (refer to Arturi and Hollender^24^ for information on prediction models for other endpoints). At the same time, as proposed in Alvarez-Mora et al.,^13^ it has been demonstrated that semiquantification advances HT-EDA by allowing for the evaluation of the contribution to total activity beyond known compounds with reference standards.

## Supplementary Material





## Notes

Chromatographic gradient for fractionation (Table S1); MS method settings (Table S2); information of target compounds (Table S3); information of steroid compounds (Table S4); information of internal standards (Table S5); workflow and settings for MZmine (Table S6); procedural blank activity (Table S7); concentrations of target compounds and activity contribution (Table S8); NTS results for ESI and APCI positive ionization modes with and without MLinvitroTox prioritization (Table S9A,B); NTS results for ESI negative ionization mode (Table S10); dose–response curves (Table S11); calculated BEQ values by BDS data evaluation tool (Table S12); heatmap of the relative bioactivity for the 104 samples measured in previous stages (Figure S1); steroid recoveries after fractionation and evaporation (Figure S2); overlapping of fractionated compounds (Figure S3); Sirius settings (Figure S4); compound Discoverer Workflow (Figure S5); dose–response curves of unfractionated and recombined extracts (Figure S6); feature reduction plots (Figure S7) (XLSX)
AR-CALUX bioassay conditions (SI.1); methodology for calculating the contributions of identified compounds (SI.2) (PDF)

## References

[1] Altenburger R., Ait-Aissa S., Antczak P., Backhaus T., Barceló D., Seiler T.-B., Brion F., Busch W., Chipman K., de Alda M. L., de Aragão Umbuzeiro G., Escher B. I., Falciani F., Faust M., Focks A., Hilscherova K., Hollender J., Hollert H., Jäger F., Jahnke A., Kortenkamp A., Krauss M., Lemkine G. F., Munthe J., Neumann S., Schymanski E. L., Scrimshaw M., Segner H., Slobodnik J., Smedes F., Kughathas S., Teodorovic I., Tindall A. J., Tollefsen K. E., Walz K.-H., Williams T. D., Van den Brink P. J., van Gils J., Vrana B., Zhang X., Brack W. (2015). Future Water Quality Monitoring — Adapting Tools to Deal with
Mixtures of Pollutants in Water Resource Management. Sci. Total Environ..

[2] Neale, P. A. ; Escher, B. I. Mixture Modelling and Effect-Directed Analysis for Identification of Chemicals, Mixtures and Effects of Concern. In A New Paradigm for Environmental Chemistry and Toxicology; Springer, 2020; pp 87–97. 10.1007/978-981-13-9447-8_7.

[3] Fatimazahra S., Latifa M., Laila S., Monsif K. (2023). Review of Hospital
Effluents: Special Emphasis on Characterization, Impact, and Treatment
of Pollutants and Antibiotic Resistance. Environ.
Monit. Assess..

[4] Verlicchi P., Galletti A., Petrovic M., Barceló D. (2010). Hospital Effluents
as a Source of Emerging Pollutants: An Overview of Micropollutants
and Sustainable Treatment Options. J. Hydrol..

[5] Almazrouei B., Islayem D., Alskafi F., Catacutan M. K., Amna R., Nasrat S., Sizirici B., Yildiz I. (2023). Steroid Hormones
in Wastewater: Sources, Treatments, Environmental Risks, and Regulations. Emerging Contam..

[6] Di
Paolo C., Ottermanns R., Keiter S., Ait-Aissa S., Bluhm K., Brack W., Breitholtz M., Buchinger S., Carere M., Chalon C., Cousin X., Dulio V., Escher B. I., Hamers T., Hilscherová K., Jarque S., Jonas A., Maillot-Marechal E., Marneffe Y., Nguyen M. T., Pandard P., Schifferli A., Schulze T., Seidensticker S., Seiler T.-B., Tang J., van der Oost R., Vermeirssen E., Zounková R., Zwart N., Hollert H. (2016). Bioassay Battery Interlaboratory
Investigation of Emerging Contaminants in Spiked Water Extracts –
Towards the Implementation of Bioanalytical Monitoring Tools in Water
Quality Assessment and Monitoring. Water Res..

[7] Brack W., Aissa S. A., Backhaus T., Dulio V., Escher B. I., Faust M., Hilscherova K., Hollender J., Hollert H., Müller C., Munthe J., Posthuma L., Seiler T.-B., Slobodnik J., Teodorovic I., Tindall A. J., de Aragão Umbuzeiro G., Zhang X., Altenburger R. (2019). Effect-Based Methods Are Key. The
European Collaborative
Project SOLUTIONS Recommends Integrating Effect-Based Methods for
Diagnosis and Monitoring of Water Quality. Environ.
Sci. Eur..

[8] Escher, B. ; Neale, P. ; Leusch, F. Bioanalytical Tools in Water Quality Assessment; IWA Publishing: London, UK, 2021; . 10.2166/9781789061987.

[9] Wernersson A.-S., Carere M., Maggi C., Tusil P., Soldan P., James A., Sanchez W., Dulio V., Broeg K., Reifferscheid G., Buchinger S., Maas H., Van Der
Grinten E., O’Toole S., Ausili A., Manfra L., Marziali L., Polesello S., Lacchetti I., Mancini L., Lilja K., Linderoth M., Lundeberg T., Fjällborg B., Porsbring T., Larsson D. J., Bengtsson-Palme J., Förlin L., Kienle C., Kunz P., Vermeirssen E., Werner I., Robinson C. D., Lyons B., Katsiadaki I., Whalley C., den Haan K., Messiaen M., Clayton H., Lettieri T., Carvalho R. N., Gawlik B. M., Hollert H., Di Paolo C., Brack W., Kammann U., Kase R. (2015). The European
Technical Report on Aquatic Effect-Based Monitoring Tools under the
Water Framework Directive. Environ. Sci. Eur..

[10] Backhaus T. (2023). Commentary
on the EU Commission’s Proposal for Amending the Water Framework
Directive, the Groundwater Directive, and the Directive on Environmental
Quality Standards. Environ. Sci. Eur..

[11] Neale P. A., Escher B. I., de Baat M. L., Dechesne M., Dingemans M. M. L., Enault J., Pronk G. J., Smeets P. W. M. H., Leusch F. D. L. (2023). Application of Effect-Based Methods
to Water Quality
Monitoring: Answering Frequently Asked Questions by Water Quality
Managers, Regulators, and Policy Makers. Environ.
Sci. Technol..

[12] Brack W., Ait-Aissa S., Burgess R. M., Busch W., Creusot N., Di Paolo C., Escher B. I., Mark Hewitt L., Hilscherova K., Hollender J., Hollert H., Jonker W., Kool J., Lamoree M., Muschket M., Neumann S., Rostkowski P., Ruttkies C., Schollee J., Schymanski E. L., Schulze T., Seiler T.-B., Tindall A. J., De Aragão
Umbuzeiro G., Vrana B., Krauss M. (2016). Effect-Directed Analysis
Monitoring of Aquatic Environments--An in-Depth Overview. Sci. Total Environ..

[13] Alvarez-Mora I., Arturi K., Béen F., Buchinger S., El Mais A. E. R., Gallampois C., Hahn M., Hollender J., Houtman C., Johann S., Krauss M., Lamoree M., Margalef M., Massei R., Brack W., Muz M. (2024). Progress Applications,
and Challenges in High-Throughput Effect-Directed Analysis for Toxicity
Driver Identification — Is It Time for HT-EDA?. Anal. Bioanal. Chem..

[14] Jonkers T., Meijer J., Vlaanderen J., Vermeulen R., Houtman C. J., Hamers T., Lamoree M. H. (2022). High-Performance
Data Processing Workflow Incorporating Effect-Directed Analysis for
Feature Prioritization in Suspect and Nontarget Screening. Environ. Sci. Technol..

[15] Zwart N., Nio S. L., Houtman C. J., de Boer J., Kool J., Hamers T., Lamoree M. H. (2018). High-Throughput Effect-Directed Analysis
Using Downscaled in Vitro Reporter Gene Assays To Identify Endocrine
Disruptors in Surface Water. Environ. Sci. Technol..

[16] Lopez-Herguedas N., González-Gaya B., Cano A., Alvarez-Mora I., Mijangos L., Etxebarria N., Zuloaga O., Olivares M., Prieto A. (2022). Effect-Directed Analysis
of a Hospital Effluent Sample
Using A-YES for the Identification of Endocrine Disrupting Compounds. Sci. Total Environ..

[17] Jonkers T., Houtman C., Van Oorschot Y., Lamoree M., Hamers T. (2023). Identification
of Antimicrobial and Glucocorticoid Compounds in Wastewater Effluents
with Effect-Directed Analysis. Environ. Res..

[18] Zwart N., Jonker W., Broek R. t., de Boer J., Somsen G., Kool J., Hamers T., Houtman C. J., Lamoree M. H. (2020). Identification
of Mutagenic and Endocrine Disrupting Compounds in Surface Water and
Wastewater Treatment Plant Effluents Using High-Resolution Effect-Directed
Analysis. Water Res..

[19] Xiao H., Brinkmann M., Thalmann B., Schiwy A., Große
Brinkhaus S., Achten C., Eichbaum K., Gembé C., Seiler T.-B., Hollert H. (2017). Toward Streamlined Identification
of Dioxin-like Compounds in Environmental Samples through Integration
of Suspension Bioassay. Environ. Sci. Technol..

[20] Hollender J., Schymanski E. L., Ahrens L., Alygizakis N., Béen F., Bijlsma L., Brunner A. M., Celma A., Fildier A., Fu Q., Gago-Ferrero P., Gil-Solsona R., Haglund P., Hansen M., Kaserzon S., Kruve A., Lamoree M., Margoum C., Meijer J., Merel S., Rauert C., Rostkowski P., Samanipour S., Schulze B., Schulze T., Singh R. R., Slobodnik J., Steininger-Mairinger T., Thomaidis N. S., Togola A., Vorkamp K., Vulliet E., Zhu L., Krauss M. (2023). NORMAN Guidance on Suspect and Non-Target Screening
in Environmental Monitoring. Environ. Sci. Eur..

[21] Sonneveld E., Jansen H. J., Riteco J. A. C., Brouwer A., van der
Burg B. (2005). Development of Androgen- and Estrogen-Responsive Bioassays, Members
of a Panel of Human Cell Line-Based Highly Selective Steroid-Responsive
Bioassays. Toxicol. Sci..

[22] Arturi K., Hollender J. (2023). Machine Learning-Based Hazard-Driven
Prioritization
of Features in Nontarget Screening of Environmental High-Resolution
Mass Spectrometry Data. Environ. Sci. Technol..

[23] Välitalo P., Massei R., Heiskanen I., Behnisch P., Brack W., Tindall A. J., Du Pasquier D., Küster E., Mikola A., Schulze T., Sillanpää M. (2017). Effect-Based
Assessment of Toxicity Removal during Wastewater Treatment. Water Res..

[24] Schulze T., Neale P. A., Ahlheim J., Beckers L.-M., König M., Krüger J., Petre M., Piotrowska A., Schlichting R., Schmidt S., Krauss M., Escher B. I. (2024). A Guidance
for the Enrichment of Micropollutants from Wastewater by Solid-Phase
Extraction before Bioanalytical Assessment. Environ. Sci. Eur..

[25] Wolf Y., Oster S., Shuliakevich A., Brückner I., Dolny R., Linnemann V., Pinnekamp J., Hollert H., Schiwy S. (2022). Improvement of Wastewater
and Water
Quality via a Full-Scale Ozonation Plant? – A Comprehensive
Analysis of the Endocrine Potential Using Effect-Based Methods. Sci. Total Environ..

[26] Chambers M. C., Maclean B., Burke R., Amodei D., Ruderman D. L., Neumann S., Gatto L., Fischer B., Pratt B., Egertson J., Hoff K., Kessner D., Tasman N., Shulman N., Frewen B., Baker T. A., Brusniak M.-Y., Paulse C., Creasy D., Flashner L., Kani K., Moulding C., Seymour S. L., Nuwaysir L. M., Lefebvre B., Kuhlmann F., Roark J., Rainer P., Detlev S., Hemenway T., Huhmer A., Langridge J., Connolly B., Chadick T., Holly K., Eckels J., Deutsch E. W., Moritz R. L., Katz J. E., Agus D. B., MacCoss M., Tabb D. L., Mallick P. (2012). A Cross-Platform
Toolkit
for Mass Spectrometry and Proteomics. Nat. Biotechnol..

[27] Schmid R., Heuckeroth S., Korf A., Smirnov A., Myers O., Dyrlund T. S., Bushuiev R., Murray K. J., Hoffmann N., Lu M., Sarvepalli A., Zhang Z., Fleischauer M., Dührkop K., Wesner M., Hoogstra S. J., Rudt E., Mokshyna O., Brungs C., Ponomarov K., Mutabdžija L., Damiani T., Pudney C. J., Earll M., Helmer P. O., Fallon T. R., Schulze T., Rivas-Ubach A., Bilbao A., Richter H., Nothias L.-F., Wang M., Orešič M., Weng J.-K., Böcker S., Jeibmann A., Hayen H., Karst U., Dorrestein P. C., Petras D., Du X., Pluskal T. (2023). Integrative Analysis
of Multimodal Mass Spectrometry Data in MZmine 3. Nat. Biotechnol..

[28] Dührkop K., Shen H., Meusel M., Rousu J., Böcker S. (2015). Searching
Molecular Structure Databases with Tandem Mass Spectra Using CSI:FingerID. Proc. Natl. Acad. Sci. U.S.A..

[29] Hoffmann M. A., Nothias L.-F., Ludwig M., Fleischauer M., Gentry E. C., Witting M., Dorrestein P. C., Dührkop K., Böcker S. (2021). Assigning Confidence to Structural
Annotations from Mass Spectra with COSMIC. bioRxiv.

[30] Schymanski E. L., Jeon J., Gulde R., Fenner K., Ruff M., Singer H. P., Hollender J. (2014). Identifying
Small Molecules via High
Resolution Mass Spectrometry: Communicating Confidence. Environ. Sci. Technol..

[31] Mohammed
Taha H., Aalizadeh R., Alygizakis N., Antignac J.-P., Arp H. P. H., Bade R., Baker N., Belova L., Bijlsma L., Bolton E. E., Brack W., Celma A., Chen W.-L., Cheng T., Chirsir P., Čirka L. ´., D’Agostino L. A., Djoumbou Feunang Y., Dulio V., Fischer S., Gago-Ferrero P., Galani A., Geueke B., Głowacka N., Glüge J., Groh K., Grosse S., Haglund P., Hakkinen P. J., Hale S. E., Hernandez F., Janssen E. M.-L., Jonkers T., Kiefer K., Kirchner M., Koschorreck J., Krauss M., Krier J., Lamoree M. H., Letzel M., Letzel T., Li Q., Little J., Liu Y., Lunderberg D. M., Martin J. W., McEachran A. D., McLean J. A., Meier C., Meijer J., Menger F., Merino C., Muncke J., Muschket M., Neumann M., Neveu V., Ng K., Oberacher H., O’Brien J., Oswald P., Oswaldova M., Picache J. A., Postigo C., Ramirez N., Reemtsma T., Renaud J., Rostkowski P., Rüdel H., Salek R. M., Samanipour S., Scheringer M., Schliebner I., Schulz W., Schulze T., Sengl M., Shoemaker B. A., Sims K., Singer H., Singh R. R., Sumarah M., Thiessen P. A., Thomas K. V., Torres S., Trier X., van Wezel A. P., Vermeulen R. C. H., Vlaanderen J. J., von der Ohe P. C., Wang Z., Williams A. J., Willighagen E. L., Wishart D. S., Zhang J., Thomaidis N. S., Hollender J., Slobodnik J., Schymanski E. L. (2022). The NORMAN
Suspect List Exchange (NORMAN-SLE): Facilitating European and Worldwide
Collaboration on Suspect Screening in High Resolution Mass Spectrometry. Environ. Sci. Eur..

[32] Sepman H., Malm L., Peets P., MacLeod M., Martin J., Breitholtz M., Kruve A. (2023). Bypassing the Identification: MS2Quant
for Concentration Estimations of Chemicals Detected with Nontarget
LC-HRMS from MS2 Data. Anal. Chem..

[33] Rogers D., Hahn M. (2010). Extended-Connectivity
Fingerprints. J. Chem.
Inf. Model..

[34] Kleinstreuer N. C., Ceger P., Watt E. D., Martin M., Houck K., Browne P., Thomas R. S., Casey W. M., Dix D. J., Allen D., Sakamuru S., Xia M., Huang R., Judson R. (2017). Development and Validation of a Computational
Model
for Androgen Receptor Activity. Chem. Res. Toxicol..

[35] Finckh S., Beckers L.-M., Busch W., Carmona E., Dulio V., Kramer L., Krauss M., Posthuma L., Schulze T., Slootweg J., Von der
Ohe P. C., Brack W. (2022). A Risk Based Assessment
Approach for Chemical Mixtures from Wastewater Treatment Plant Effluents. Environ. Int..

[36] Huang X., Cang X., Liu J. (2019). Molecular
Mechanism of Bisphenol
A on Androgen Receptor Antagonism. Toxicol.
in Vitro.

[37] van
der Burg B., Winter R., Man H., Vangenechten C., Berckmans P., Weimer M., Witters H., van der
Linden S. (2010). Optimization and Prevalidation of the in Vitro AR CALUX Method to
Test Androgenic and Antiandrogenic Activity of Compounds. Reprod. Toxicol..

[38] Xiang T., Shi C., Guo Y., Zhang J., Min W., Sun J., Liu J., Yan X., Liu Y., Yao L., Mao Y., Yang X., Shi J., Yan B., Qu G., Jiang G. (2024). Effect-Directed Analysis
of Androgenic Compounds from Sewage Sludges
in China. Water Res..

[39] Huanyu T., Jianghong S., Wei G., Jiawei Z., Hui G., Yunhe W. (2022). Environmental Fate and Toxicity of Androgens: A Critical Review. Environ. Res..

[40] Phan M. A. T., Gibson E., Golebiowski B., Stapleton F., Jenner A. M., Bucknall M. P. (2022). Analysis of Sex
Steroids in Human
Tears Using LC-MS and GC-MS: Considerations and Developments to Improve
Method Sensitivity and Accuracy. Exp. Eye Res..

[41] Topor L. S., Asai M., Dunn J., Majzoub J. A. (2011). Cortisol
Stimulates
Secretion of Dehydroepiandrosterone in Human Adrenocortical Cells
through Inhibition of 3betaHSD2. J. Clin. Endocrinol.
Metab..

[42] Tölgyesi A., Verebey Z., Sharma V. K., Kovacsics L., Fekete J. (2010). Simultaneous Determination of Corticosteroids,
Androgens,
and Progesterone in River Water by Liquid Chromatography-Tandem Mass
Spectrometry. Chemosphere.

[43] AID 588515—qHTS assay for small molecule agonists of androgen receptor signaling; PubChem. https://pubchem.ncbi.nlm.nih.gov/bioassay/588515 (accessed 08 26, 2024).

[44] AID 1259387—qHTS assay to identify small molecule agonists of the androgen receptor (AR) signaling pathway in the presence of an antagonist: Summary; PubChem. https://pubchem.ncbi.nlm.nih.gov/bioassay/1259387 (accessed 08 26, 2024).

[45] Cha J., Hong S., Lee J.-H., Gwak J., Kim M., Kim T., Hur J., Giesy J. P., Khim J. S. (2021). Novel Polar AhR-Active
Chemicals Detected in Sediments of an Industrial Area Using Effect-Directed
Analysis Based on in Vitro Bioassays with Full-Scan High Resolution
Mass Spectrometric Screening. Sci. Total Environ..

[46] Andriulli A., Arrigoni A., Gindro T., Karbowiak I., Buzzetti G., Armanini D. (2004). Canrenone and Androgen
Receptor-Active
Materials in Plasma of Cirrhotic Patients during Long-Term K-Canrenoate
or Spironolactone Therapy. Digestion.

[47] Long B. J., Grigoryev D. N., Nnane I. P., Liu Y., Ling Y.-Z., Brodie A. M. (2000). Antiandrogenic
Effects of Novel Androgen Synthesis
Inhibitors on Hormone-Dependent Prostate Cancer. Cancer Res..

[48] Wang H., Ding Z., Shi Q.-M., Ge X., Wang H.-X., Li M.-X., Chen G., Wang Q., Ju Q., Zhang J.-P., Zhang M.-R., Xu L.-C. (2017). Anti-Androgenic
Mechanisms of Bisphenol A Involve Androgen Receptor Signaling Pathway. Toxicology.

[49] Ochnik A. M., Moore N. L., Jankovic-Karasoulos T., Bianco-Miotto T., Ryan N. K., Thomas M. R., Birrell S. N., Butler L. M., Tilley W. D., Hickey T. E. (2014). Antiandrogenic Actions
of Medroxyprogesterone
Acetate on Epithelial Cells within Normal Human Breast Tissues Cultured
Ex Vivo. Menopause.

