# Supplementary material for: High-Throughput Effect-Directed Analysis of Androgenic Compounds in Hospital Wastewater: Identifying Effect Drivers through Non-Target Screening Supported by Toxicity Prediction
Source: Environ Sci Technol. 2025 Jan 8;59(45):24513–25. doi: 10.1021/acs.est.4c09942 (PMC12631978; doi:10.1021/acs.est.4c09942)
Supplement: Supplementary file 2 [file es4c09942_si_002.pdf]

Supporting Information: High-Throughput Effect-Directed Analysis of Androgenic Compounds in Hospital Wastewater: Identifying Effect Drivers through Non-Target Screening Supported by Toxicity Prediction

Iker Alvarez-Mora[1,2,*], Aset Muratuly[1,2], Sarah Johann[3], Katarzyna Arturi[4], Florian Jünger[3], Carolin Huber[1], Henner Hollert[3,5,6,7], Martin Krauss[1], Werner Brack[1,3], and Melis Muz[1].

* Corresponding author: **iker.alvarez-mora@ufz.de**

1 Department Exposure Science, Helmholtz Centre for Environmental Research - UFZ, 04318 Leipzig, Germany

2 Research Centre for Experimental Marine Biology and Biotechnology (PIE), University of the Basque Country (UPV/EHU), 48620 Plentzia, Basque Country, Spain

3 Department of Evolutionary Ecology and Environmental Toxicology, Goethe University Frankfurt, 60438 Frankfurt am Main, Germany

4 Department of Environmental Chemistry, Swiss Federal Institute of Aquatic Science and Technology (Eawag), 8600 Dübendorf, Switzerland

5 Department Environmental Media Related Ecotoxicology, Fraunhofer Institute for Molecular Biology and Applied Ecology (IME), 57392 Schmallenberg, Germany

6 LOEWE Centre for Translational Biodiversity Genomics (LOEWE-TBG), 60325 Frankfurt am Main, Germany

7 Kompetenzzentrum Wasser Hessen, 60438 Frankfurt am Main, Germany

Summary: *3 pages / 2 additional texts (.pdf) + 12 tables & 7 Figures (.xlsx)*

Table of Contents:

.pdf:

SI.1: AR-CALUX bioassay conditions

SI.2: Contribution of identified compounds to fraction activity

.xlsx:

Table S1: Chromatographic gradient for fractionation.

Table S2: MS method settings.

Table S3: Information of target compounds.

Table S4: Information of steroid compounds.

Table S5: Information of internal standards.

Table S6: Workflow and settings for MZmine.

Table S7: Procedural blank activity.

Table S8: Concentrations of target compounds & activity contribution.

Table S9A & S9B: NTS results for ESI and APCI positive ionization modes with and without MLinvitroTox prioritization.

Table S10: NTS results for ESI negative ionization mode.

Table S11: Dose-response curves.

Table S12: Calculated BEQ values by BDS data evaluation tool.

Figure S1: Heatmap of the relative bioactivity for the 104 samples measured in previous stages.

Figure S2: Steroid recoveries after fractionation and evaporation.

Figure S3: Overlapping of fractionated compounds.

Figure S4: Sirius settings.

Figure S5: Compound Discoverer Workflow.

Figure S6: Dose-response curves of unfractionated and recombined extracts.

Figure S7: Feature reduction plots.

SI.1: AR-CALUX bioassay conditions

The Chemically Activated LUciferase eXpression (CALUX) assay (AR-CALUX®) was performed to evaluate androgenic activity of the sample and its fractions. The assay was performed on human osteosarcoma U2-OS cells purchased from Biodetection Systems (BDS, The Netherlands) which were transfected with a firefly luciferase reporter gene, coupled to the human androgen receptor (AR) as detailed in Sonneveld et al. (2005)and Wolf et al. (2022). Cells were maintained in DMEM/F12 (1:1) medium (with phenol red and Glutamax), supplemented with 7.5% fetal calf serum (FCS), non-essential amino acids, and a penicillin-streptomycin solution (10,000 units, 10 mg/mL) in 75 cm² flasks at 37 °C, 97% humidity and 5% CO2. Reaching 90% confluency in the flasks, cells were passaged or used for assay procedure.

For the assay, U2-OScells were seeded either in 96- (for raw and recombined samples, (Di Paolo et al. 2016)) or 384-well plates (for fractions, (2018)) with density of 100,000 cells/mL or 200,000 cells/mL according to OECD No. 458 (2023) and Di Paolo et al (2016) and Zwart et al. (2018) respectively. Experiments in 384-well plates were performed due to reduced sample volume available for the fractions. Cells were incubated for 24 h at 37°C, 95% humidity, and 5% CO2 in the assay medium (DMEM/F12, w/o phenol red, supplemented with 7.5% stripped FCS and Minimum Essential Medium). The medium was then replaced with the exposure medium, consisting of assay medium supplemented with the sample extract or its fractions prepared in a 1:2 serial dilution (0.1% - 0,2% solvent DMSO), as well as the positive control (Dihydrotestosterone, DHT) dilution series (1 pM -100 nM). The final volume of the exposure medium was 100 μL for 96-well plates, and 34 μL for 384-well plates. Each plate contained a full dilution series of the reference compound DHT in triplicates and sample dilutions tested in triplicates or as single wells for individual fractions. The plates were incubated for 24 hours, after which the cells were lysed using 30 µL of lysis buffer (25 mM TRIS, 2 mM 1,4-DTT, 2 mM CDTA disodium salt, 10% glycerol, 1% Triton X-100). 30 µL (96-well plates) or 17 µL (384-well plates) of luciferin substrate (20 mM tricine, 1.07 mM $(MgCO_3)_4Mg(OH)_2 \cdot 5H_2O$, 0.1 mM EDTA, 1.5 mM 1,4-DDT, 530 μM 21 d-luciferin, 5,49 mM ATP) were added to the lysate, and the luciferase activity was measured using a Tecan Spark multimode microplate reader (Tecan Trading AG, Switzerland). The validity of each plate was evaluated based on EC50 range (100 pM –

1000 pM) and R^2 (≥ 0.98) of DHT calibration curve, as well as induction factor (≥ 5) and z-factor (>0.5) as recommended by BDS. Bioanalytical equivalents (BEQ) were calculated based on the DHT calibration curve. Additionally, dilution series of the sample extract, recombined sample, and active fractions were prepared to build the concentration-response curves and determine the EC10 values. EC10 values were used instead of conventional EC50 for BEQ_{bio} calculations (See section 2.7). This is a common practice when using gene-reporter assays to avoid interference from cytotoxicity (Escher et al., 2021).

SI.2: Contribution of identified compounds to fraction activity

The results from the (semi-)quantification and dose-response curves were used to assess the contribution of the compounds. Relative potency values (REP) values reported in the literature were used for these estimates, or EC50 values were employed to calculate the REP where appropriate:

$$REP = \frac{\mathrm{EC_{50}\ (DHT)}}{\mathrm{EC_{50}\ (compound)}}$$

Bio-analytical equivalents (BEQbio) of the sample extract and its fractions were calculated as the ratio of the EC10 values of the sample and DHT:

$$BEQ_{bio} = \frac{\mathrm{EC_{10}\ (DHT)}}{\mathrm{EC_{10}\ (sample)}}$$

The bio-analytical equivalents of the identified compounds in the fraction were calculated as the sum of the product of the concentration of the compound and its REP value:

$$BEQ_{chem} = \sum_{i}^{n} REP_i \ x \ C_i$$

where n is the number of identified compounds in the fraction, Ci is the concentration of the compound, and REPi is the relative effect potency of the compound. These results were compared to the total BEQ of the fraction to evaluate the contribution of the identified compounds to the total androgenic activity of the fraction.

$$Contribution\ (\%) = \frac{\mathrm{BEQ}_{chem}}{\mathrm{BEQ_{bio}}} x\ 100$$
